# Recreation impact on the early establishment of dune-building grasses *Elytrigia juncea* and *Ammophila arenaria* on the beach

**DOI:** 10.1007/s11852-025-01101-5

**Published:** 2025-01-24

**Authors:** S.J. van Rosmalen, J.-M. Homberger, M.J.P.M. Riksen, J. Limpens

**Affiliations:** 1 Plant Ecology and Nature Conservation Group, Wageningen University and Research, Wageningen, the Netherlands; 2 Soil Physics and Land Management Group, Wageningen University and Research, Wageningen, the Netherlands

**Keywords:** Dune development, Recreation, Anthropogenic impacts, Plant establishment

## Abstract

Sandy shores serve multiple ecosystem services, including recreation. To what extent these services can coexist is unclear, especially given increasing stressors such as rising sea levels and urbanization. We investigated the effect of recreational pressure on the establishment of two dune building grass species representative for European beaches (*Ammophila arenaria and Elytrigia juncea*). We conducted a field introduction experiment with seeds and rhizomes (diaspores) of both species along an anthropogenic pressure gradient on the upper beach of the Dutch barrier Island of Terschelling. Across two beach sites 300 plots were set out following a randomised block design with 4 factorial treatments (species*diaspore). Local plant material was collected. Plots were left unmarked to enable undisturbed recreation. Establishment success was monitored by counting the number of emerged shoots per plot at regular intervals across the growing season of 2022. To control for environmental drivers, we included the environmental variables: soil moisture, bed level change, and distance to the sea. We found that establishment success increased significantly with longshore distance from the beach entrance, irrespective of species or diaspore type. This effect was especially strong within the first 100 m, where plants did emerge from seeds or rhizomes but progressively died over the summer. Establishment success was further constrained by changes to the beach bed level and distance to the sea. Our results indicate that recreational pressure can constrain dune development on the upper beach. This implies trade-offs between beach functions, that should be considered when designing sandy coastal areas.

## Introduction

Coastlines are densely populated (Small and Nicholls 2003) and are often heavily impacted by human activities. Accounting for 31% of the world’s coastlines (Luijendijk et al. 2018), sandy coasts provide many ecosystem services that are associated with sand dunes in different stages of development. These services include natural flood protection, drinking water provision, high biodiversity, and recreation (Everard et al. 2010). To what extent these ecosystem services can coexists or are mutually exclusive remains unclear. Insights in potential trade-offs are important, especially given increased stressors to the vegetation like more frequent and more extreme storm surges under climate change (Oppenheimer et al. 2019) and increased recreational activities at elevated temperatures (Coombes and Jones 2010).

Dune development and growth rely on a bio-geomorphic process where sand accumulates as vegetation traps it. As vegetation grows, it can outgrow the sand that buries it, creating a positive feedback loop that further enhances dune formation (Arens et al. 2013; Hesp et al. 2019; Montreuil et al. 2013; Reijers et al. 2019). As a result, the probability and rate of dune formation is co-determined by the traits of the plants initiating their formation. For example, the higher salt-tolerance of dune building grass species *Elytrigia* juncea enables initiation of dunes at lower elevations (c. 1.2 m above mean sea level) than that of the grass species *Ammophila arenaria* (c. 1.6 m above mean sea level) (Nolet and Riksen 2019; Van Puijenbroek et al. 2017; van Puijenbroek, Teichmann et al. 2017). Also, differences in the clonal expansion strategy and growth-form between these species are reflected in dune shape and growth rate (Lammers et al. 2023). *Elytrigia* typically creates lower and more elongated dunes with sparse vegetation cover while *Ammophila* creates dunes that are more cone shaped with high vegetation cover (Hesp et al. 2019; Zarnetske et al. 2012). Whether the physiological and ecological differences between these species result in different sensitivity to recreation pressure is currently unknown and may co-determine the potential for dune initiation on anthropogenic beaches.

The potential distribution of dune-building grasses on the beach (also referred to as accommodation space) is constrained by strong environmental gradients, which are determined by drivers such as storm surges, wave action, inundation, and salinity (Nolet and Riksen 2019; van Puijenbroek, Limpens et al. 2017; van Puijenbroek, Teichmann et al. 2017). In addition, sediment accretion and erosion are crucial factors that impact plant growth and development (Maun 2009). Plants on anthropogenic coasts face an additional source of stress associated with the impact of recreation and beach management (Kelly 2014), potentially further reducing the accommodation space. Trampling, driving, and management practices all affect plant growth, with the impact depending on the type and intensity of the activities (Brown and McLachlan 2002; Kelly 2014). In both embryonic dunes, foredunes and inland dunes, trampling (by walking) has negative effects on the vegetation abundance and diversity (Andersen 1995; Brown and McLachlan 2002; Ciccarelli 2014; Hylgaard 1980; Šilc et al. 2017; Tzatzanis et al. 2003). In foredunes even low levels of trampling (200 passages in 4 months) can cause a reduction in the vegetation cover by 50% while for embryonic dunes, the sensitivity of adult vegetation to trampling seems to be higher than for other dune types (Šilc et al. 2017). Given that adult plants are generally sturdier with more reserves than young plants, it is likely that young shoots, just emerging from rhizomes or seeds, may be more sensitive still. For example, while a maximum tolerance of 100 cm of burial during the growing season has been reported for adult *Ammophila* (Nolet et al. 2018), this was only 40 cm for rhizome pieces (Konlechner et al. 2013), and 6 cm for shoots emerging from seeds (Bonte et al. 2021; Lim 2011). Whether the high sensitivity of shoots emerging from seeds and rhizomes is also reflected in a higher sensitivity to recreation pressure is unclear.

In this research we explored how beach recreation affects the establishment of dune-building grasses on the upper beach. We hypothesized that: (1) recreation pressure decreases plant establishment success, since trampling is known to negatively impact vegetation (Andersen 1995; Šilc et al. 2017), (2) Ammophila is more sensitive to recreation pressure than pioneer species Elytrigia, as later-successional species tend to be more sensitive to disturbances then pioneer species (Andersen 1995), (3) recreation pressure affects establishment from seeds more than establishment from rhizomes because seeds have a lower tolerance to disturbances than rhizomes (Bonte et al. 2021; Konlechner et al. 2013; Lim 2011). To test these hypotheses, we performed a field introduction experiment across an anthropogenic pressure gradient on the upper beach at two locations in the Netherlands. The dune building grasses *Ammophila arenaria* and *Elytrigia juncea* were introduced as seeds and rhizomes and their emergence from the soil was documented across the growing season. To control for drivers other than recreation pressure, we also monitored environmental variables soil moisture, bed level change, and distance to the sea. Both grass species have a wide geographical distribution and their key role in initiating the first small, vegetated, dunes (also referred to as embryo dunes, nebhka dunes or insipient fore dunes) is well documented across north-western Europe (Van Puijenbroek et al. 2017).

## Materials and methods

### Research sites

**Fig. 1:**
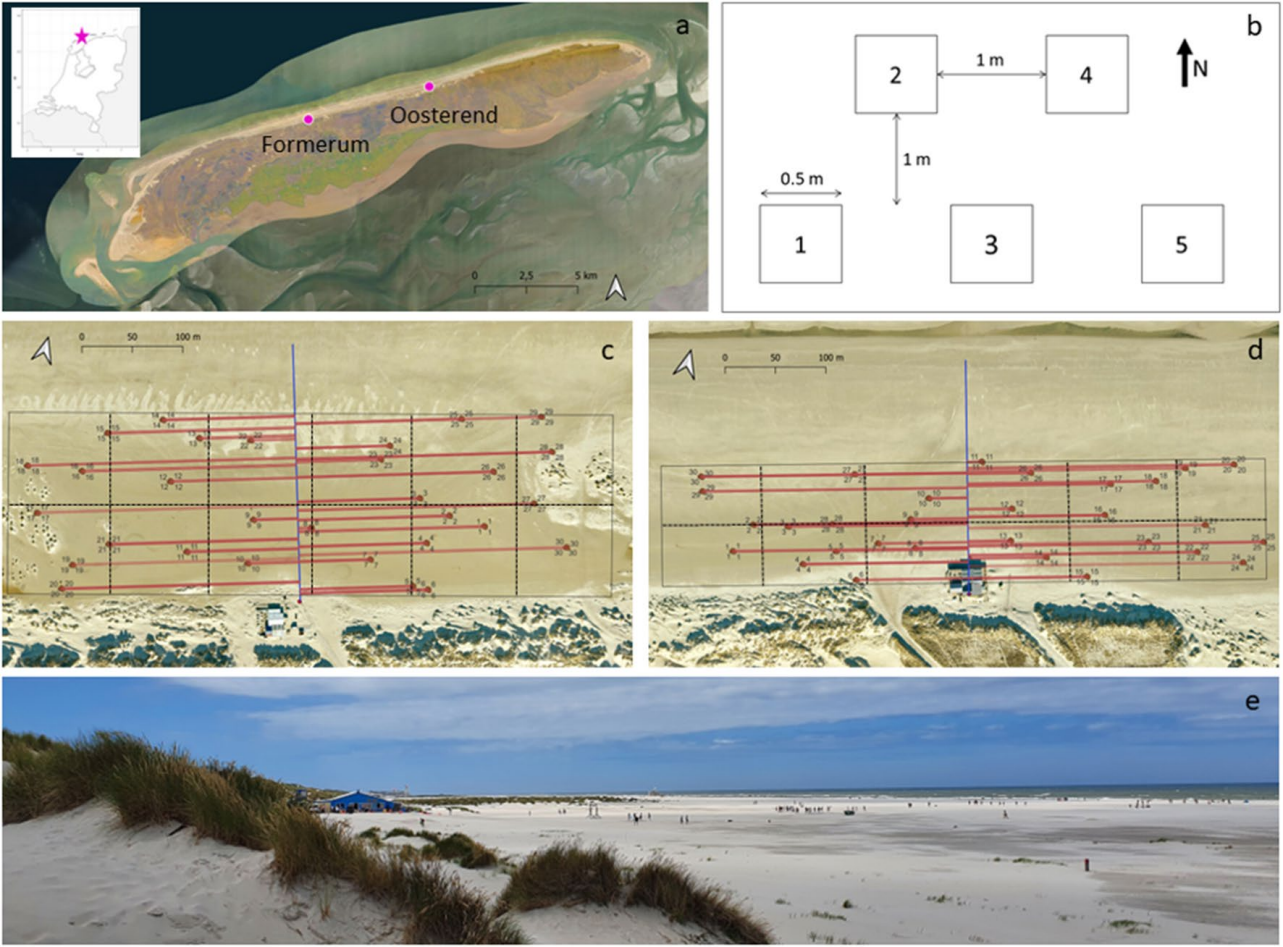
(A) The locations of the experimental sites on the barrier island Terschelling, and the location of Terschelling within the Netherlands. (B) Overview of setup of the plots within a block. (C) Formerum site overview (the beach entrance is to the right of the building, to the left is a blowout) and (D) Oosterend site overview. The black line outlines the research area at each site, in brown the plots, in blue the pathway, in red the distance to the path, black dashed lines indicate the blocks in which beach visitors were counted. (E) Overview of Formerum beach from foredune to sea with beach restaurant and beach visitors

We assessed the impact of recreation pressure on the establishment of dune-building grasses at two beaches (lat.: 53.40878, long.: 5.298435 and lat.: 53.42321, long.: 5.386428) on the Dutch barrier island of Terschelling near the villages Formerum and Oosterend (Fig. 1A). We selected these sites, for both have natural wide beaches (approximately 280 m at Formerum and 200 m at Oosterend from the dune toe to the high-water line) which are not mechanically cleaned. The beaches are mostly bare but support some sparse vegetation and little embryo dune development. Additionally, recreational facilities are present at both sites. These consist of parking facilities at the inland side of the beach entrance and restaurants on the seaside at the dune toe. The experimental period, lasting between March 2022 and February 2023, covered a warm and dry summer and a mild, storm free winter (Huiskamp 2023).

###  Experimental design

In March 2022, we created an introduction experiment to assess the establishment success of dune-building species (*Ammophila* and *Elytrigia*) at two beaches. Along the shore, plots were placed with increasing distance to the beach entrance, reflecting a gradient in recreation intensity. In cross-shore direction, plots extended from the dune foot to the approximate vegetation limit near the high-tide mark. The plots were placed between 1.7 and 3.3 m above mean sea level. Per site, plots were arranged in 30 groups (experimental blocks) of 5 plots, resulting in 300 plots in total across both sites (Fig. 1C, D). Each block contained 5 plots, 4 with different vegetation treatments and one blanco control. The plots were placed on bare sand with a similar slope within a block at minimally 1 m distance from existing vegetation. The control plot was included to correct for natural plant establishment. The plot assignment within each block was done at random (Fig. 1B). The plots (0.5 × 0.5 m) were located at least 1-meter apart and were georeferenced by means of a Real-Time Kinematic Positioning System (RTK, manufacturer: Topcon) and left unmarked to enable undisturbed recreation. To ensure a spatially representative and random placement of blocks across the beach area, we used doubly balanced sampling, with equal inclusion probabilities. In doubly balanced sampling a random sample is spread in geographical space to minimize spatial autocorrelation and auxiliary information (e.g. covariates) is used for balancing. This should improve the estimate of the variable of interest (r package “BalancedSampling”) (Grafström et al. 2024; Grafström and Tillé 2013). For the spreading and balancing of the random sample, we used variables derived from digital terrain models (DTM). These models are published by Rijkswaterstaat, the executive agency of the Ministry of Infrastructure and Water Management and are available under a Creative Commons Zero (CC0) license (last access: 22.11.2023). As spreading variables, we used the centre coordinates of the raster pixels (x and y). As balancing variables, we used the topographical wetness index, the height above sea level in 2021, the average yearly change in height (2016–2021), and the distance from beach entrance since we anticipated these variables to correlate with the establishment success of dune-building grasses. The most recent digital terrain model (2021) served as an estimate of the height above sea level. Based on the same model, we also calculated the topographical wetness index with the SAGA GIS wetness algorithm (Conrad et al. 2015). The average yearly change in height was derived by calculating the average of the difference in heights between two consecutive years (Eq. 1):

1 $$\;DTM_{\triangle height}\;=\;\left(DTM_{2017}\;-\;DTM_{2016}\;+\;DTM_{2018}\;-\;DTM_{2017}\;+\;DTM_{2019}\;-\;DTM_{2018}\;+\;DTM_{2020}\;-\;DTM_{2019}\;+\;DTM_{2021}\;-\;DTM_{2020}\right)^{/5}\;$$

Finally, the distance from beach entrance was calculated as the normalized distance between each raster pixel (0.5 × 0.5 m) to the main beach entrance with 1 being the pixel closest to the entrance.

### Plant material

The plant material for the introduction experiment was collected locally from dune-building grasses *Ammophila* (*Calamagrostis arenaria (L.)* and *Elytrigia* (*Elymus farctus boreoatlanticus (L.) Roth*,* Elymus junceiformis*,* Elytrigia junceiformis*). We introduced two types of diaspores, namely seeds and rhizomes. The seeds were manually collected in August 2021 by cutting off the inflorescence and storing them at ambient outside temperatures and humidity. *S*eeds of *Elytrigia* were not further pre-processed. Whereas the *Ammophila* seeds were obtained from the inflorescence by mechanical threshing and manual sieving. When present, the husks were not removed from the seeds in order to mimic natural dispersion of seeds (Huiskes 1979). The rhizomes were collected locally from adult plants up to 2 days before the creation of the plots and were stored at ambient soil temperatures. Harris and Davy (1986) found an increased shoot emergence success from multiple-node rhizomes pieces, therefore we cut the rhizome pieces to have two clear nodes and considered two potential plants to grow per rhizome piece. The combination of a species and diaspores resulted in 4 treatments: *Ammophila* rhizomes, *Ammophila* seeds, *Elytrigia* rhizomes, and *Elytrigia* seeds. The treatment plots containing rhizomes had 20 rhizome pieces (40 nodes). This was the maximum number of rhizomes that fitted in the plot. For the seed plots, the number of seeds was based on the results of a preliminary test. This test showed especially low germination of *Ammophila*, therefore we decided to increase the number of seeds per plot for this treatment. The number of seeds was determined using the average seed count of 1 gram of material of *Ammophila* or 10 g of *Elytrigia* multiplied by the number of grams per plot. This resulted for *Ammophila* and *Elytrigia* respectively, in 1055 (5.00 ± 0.05 g) and 214 (9.00 ± 0.05 g) seeds per plot. All diaspores were planted at a depth of approximately 2 cm. From this depth plant establishment is possible and the material is not immediately carried away (Bonte et al. 2021; Harris and Davy 1986a; Hilton and Konlechner 2011; Lammers et al. 2024).

### Measurements

Beach visitors were counted to verify the gradient in recreation pressure with increasing distance to the beach entrance. This was done for 4 days during peak beach season (July and August). At each site counting was done across two days with dry and sunny weather conditions, including a weekend day. The sites were divided into 12 blocks of equal size (Formerum: 0.9 ha, Oosterend: 0.6 ha, Fig. 1C, D). In each of the blocks all visitors were counted 6 times for 15 min. The counting was done by a single observer situated on the foredune with binoculars. The corners of these fields were indicated by sticks as a visual reference point. Counting confirmed the existence of an along-shore anthropogenic pressure gradient. Furthermore, it was observed that most visitors moved in a straight line from the beach entrance to the sea. Therefore, we expressed recreation pressure as ‘distance to the pathway’ for further analyses. Pathways were defined as a straight line from beach entrance to the sea (Fig. 1C, D, E). Because the Oosterend site has two entrances with a beach restaurant in between them, the middle between the two entrances was chosen as the main pathway. The distance to the pathway was calculated using QGIS (3.22) (QGIS Association 2022), ranging between 11 m and 294 m per plot.

To test the effect of anthropogenic pressure and the environmental drivers on vegetation, three vegetation monitoring rounds were completed during the growing season of 2022 in May, August, and October. An additional fourth monitoring moment was added in January 2023 to see if the vegetation patterns changed after the end of the tourist season. For this last monitoring moment, we only visited blocks in which vegetation establishment had been observed during any of the prior rounds. At each monitoring moment we counted the number of emerged shoots in each plot. These numbers were later corrected for the control plot of the same block, by subtracting the number of shoots in the control from the number of shoots in the treatment plots. Across measurement moments, only few control plots had shoots in them (13 out of 226) containing a maximum of 8 shoots per plot (mean = 0.18 shoots). In comparison, 71–101 plots out of 226 treatment plots had shoots. When a control had more shoots than a treatment the shoot count of the treatment was assumed to be zero. For visualization purposes, we also calculated the relative plant success by dividing the corrected shoot number per plot by the number of introduced diaspores, assuming each of the individual diaspores could potentially establish into a viable shoot.

To correct for the effects of other drivers on plant establishment and growth, we also monitored the elevation, change in bed level, soil moisture, soil salinity, and distance to the sea per plot. The elevation was measured for the NW and SE corners of each plot using an RTK at the plot creation and was remeasured at each monitoring round. The change in bed level was calculated by subtracting the initial elevation from the measured height at each monitoring moment based on the averages of both corners. To measure soil moisture and salinity a W.E.T. sensor kit with a HH2 moisture meter and a WET-2 sensor (manufacturer: DELTA-T Devices LTD) was inserted in the middle of each plot. For soil salinity we found EC values between 0 and 45.3 mS/cm across the entire experiment (May – January). However, the values did not exceed 4.8 mS/cm during the growing season (May – October). As this is well below the threshold which affects both *Elytrigia* or *Ammophila* (van Puijenbroek, Teichmann et al. 2017), salinity was not further considered. Distance to the sea was calculated similarly to distance to the pathway, by means of QGIS in reference to the high-water line.

### Statistical data analyses

All data analyses and visualisation were performed using R (4.3.1) (R Core Team 2024). For the data organization we used the packages “openxlsx” (Schauberger et al. 2023) and “dplyr” (*Package “Dplyr”: A Grammar of Data Manipulation* 2023). The packages “ggplot2” (Wickham et al. 2024), “gratia”(Simpson 2024), and “ggpubr” (Kassambara 2023) were used to visualise the data.

We used a Kruskal-Wallis and a pairwise Wilcoxon ranks sum test (Package “stats”) to test the differences in visitor numbers across the beach sections and differences in overall plant success between treatments. To test if anthropogenic pressure plays a role in the emerged number of shoots of dune-building grasses, we used a generalized additive model (GAM) (package “mgcv” (Wood 2023). Different models were created for the monitoring moments May, August, and October, here we expected the effect of recreation pressure to be most noticeable. In winter recreation on Dutch beaches is typically limited, therefore we did not consider the last monitoring moment. The GAM model predicts the average number of shoots in a plot. As explanatory variables we used: treatment, path distance, sea distance, soil moisture, bed level change, and several interactions (for the model equation see Appendix Eq. 2). All selected variables were also included as interaction effects with path distance to understand if anthropogenic impacts occur by themselves or if instead the interaction between environmental and anthropogenic impacts are driving the patterns. Additionally, the interaction between sea distance and treatment was included to test for differences between the species in their ability to cope with impact from the sea which has been observed in adult vegetation (Nolet and Riksen 2019; van Puijenbroek, Limpens et al. 2017). Block and study area were included as random effect to account for spatial autocorrelation between plots of the same block and site-specific effects. The statistical model assumptions of the GAM model were checked by means of simulated model residuals using the package “DHaRMa” (Hartig and Lohse 2022). We checked the concurvity at a concurvity level of 0.5 (Ramsay et al. 2003) of all variables and combinations. Based on this, elevation was removed from the model in favour of sea distance, as the later was a better predictor. A negative binominal distribution was selected because our data is count data (Zuur et al. 2009) and “REML” was used as smoothness selection method (Wood 2010). For model selection the double penalty approach by Marra and Wood (2011) was used. This is a “shrinkage” method for automatic term selection. Testing for homogeneity of variance showed no significant quantile deviations for the simulated residuals of individual predictors. Other model assumptions showed there were no violations for dispersion, outliers, remaining spatial autocorrelation or zero-inflation.

## Results

### Overall plant success

The relative shoot emergence success ranged between 0% and 173% of the introduced seeds and rhizomes, with an overall mean success of 5.6% (SE = 0.02). Values varied between species and diaspore type, with *Elytrigia* scoring on average higher than *Ammophila* and rhizomes treatments higher than those with seeds. Across all measurement moments, *Ammophila* rhizomes had a mean shoot emergence success of 7.3% (SE = 0.10), *Ammophila* seeds of 1.1% (SE = 0.02), *Elytrigia* rhizomes 5.6% (SE = 0.05) and *Elytrigia* seeds 8.2% (SE = 0.07) (percentages per measurement moment see Appendix Table 2). Emergence of seedlings from seeds and new shoots from rhizomes occurred throughout the studied period.

### Anthropogenic pressure

**Fig. 2:**
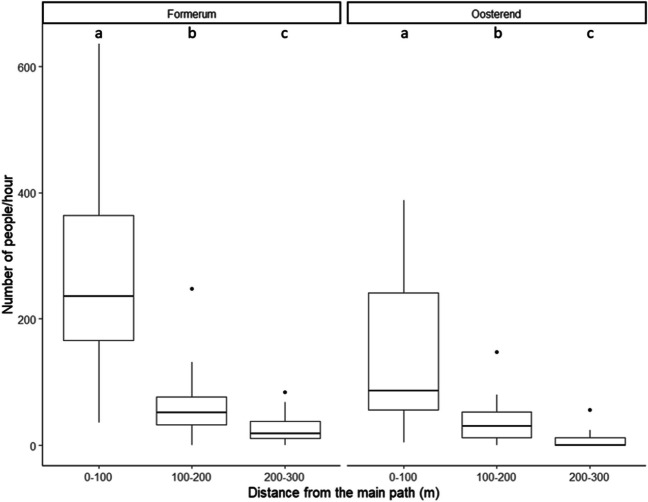
The number of people counted per 100-meter along shore interval from the main beach path (the path going straight from the entrance to the sea). The middle line indicates the median and the box the 25th −75th quantile. The letters indicate the significant differences in the counted number of people for each site per distance group. The data was collected during 36 (15 min) counting moments per site (Formerum and Oosterend) across two days with good weather in the main tourist season

At Formerum a total of 2234 visitors (207 visitors/ha) were counted and at Oosterend 1134 (158 visitors/ha) across two days per location. The number of counted visitors significantly decreased in long-shore direction from the beach entrance at both locations (Formerum *p* > 0.00, Oosterend *p* > 0.00, Appendix Tables 3 and 4). 76.2% of the visitors were observed in the 0–100 m range compared to 18.2% in the 100–200 m range and 5.6% in the 200–300 m range. This confirms the existence of an anthropogenic pressure gradient in longshore direction away from the entrance and the main path (Fig. 2).

**Fig. 3:**
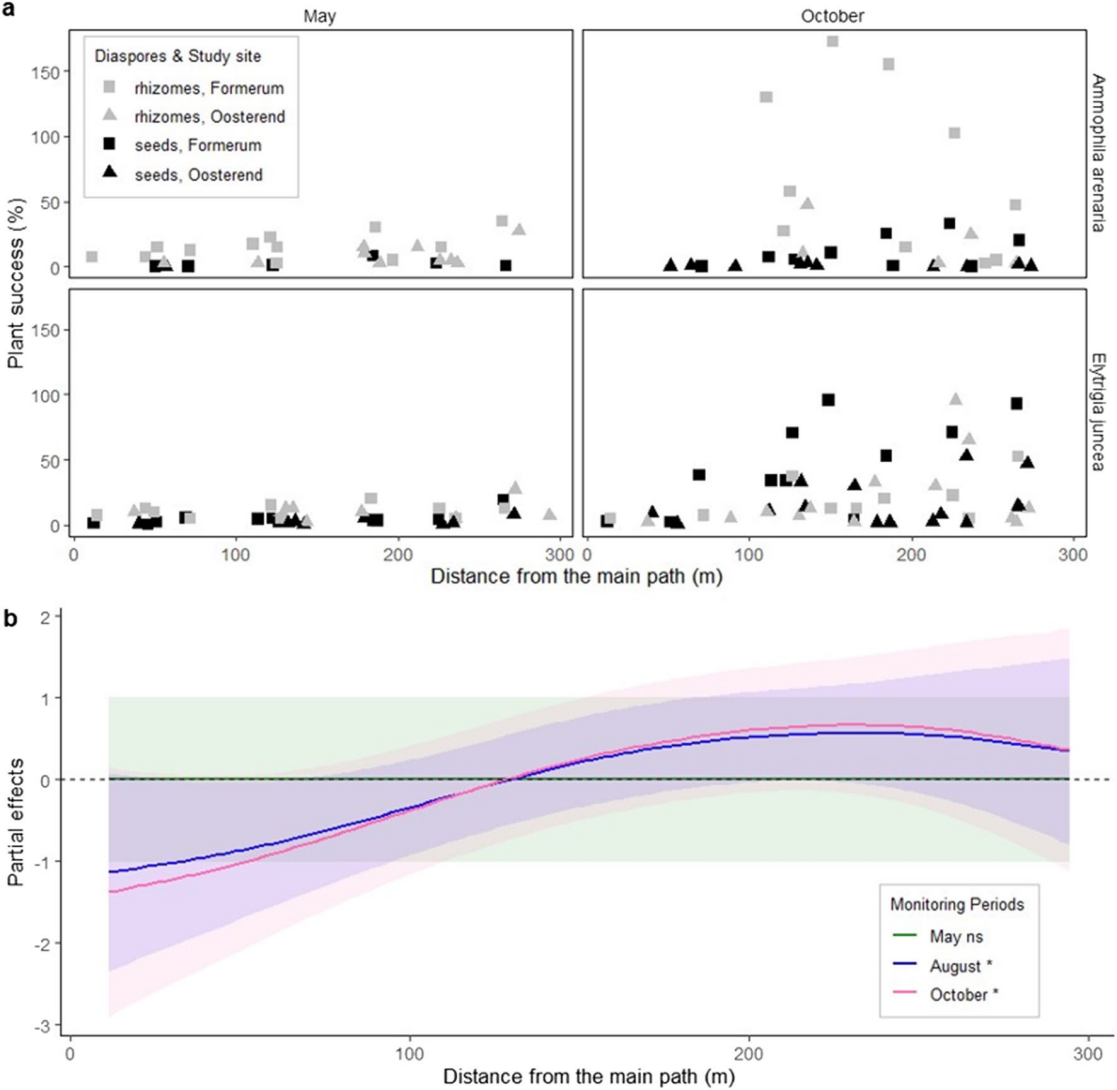
(A) Presence data of the relative plant success (shoot emergence relative to the number of introduced diaspores) versus distance from the main path (m), per species and diaspore and location for the May and October monitoring moments (before and after peak tourism season). The relative plant success is based on the shoots relative to the amount of introduced material per plot, it is possible to have multiple shoots from the same rhizome piece or seed rather than just one and thus a higher than 100% success. (B) Modelled relationships of the partial effects of the smoothers relative to the mean effect. In green May, blue August and pink October. Above the horizontal dotted line at the 0 indicates a positive effect, below a negative effect of the path distance on plant numbers. The shaded area indicate the 95% confidence interval

Shoot emergence changed significantly along the gradient in anthropogenic pressure, with the impact becoming more evident over time (Fig. 3A, for all monitoring moments see Appendix Fig. 5). In May, path distance, and thus anthropogenic pressure, did not yet significantly explain shoot emergence (Table 1, Appendix Tables 5, 6 and 7). However, as the growing and recreation season progressed this changed. Anthropogenic pressure significantly affected the number of shoots in both August and October. The curves of the smoother functions show that there is a negative effect of path distance on the shoot counts at lower distances and a positive effect at greater distances (Fig. 3B). Moreover, the difference between the moments suggests that the impact might be even stronger in October than in August. At both moments, however, the tipping point from a negative to a positive effect was at around 120 m distance from the main pathway. This is in line with our findings about anthropogenic pressure which is the highest in the first 100 m. By January, shoot numbers had decreased, but the pattern in shoot emergence and survival imposed by anthropogenic pressure was sustained (Appendix Fig. 5). While our statistical model suggests that species and diaspore seemed to respond similarly to path distance, the observed distribution of the surviving seedlings and shoots suggested a slight difference between the treatments and their ability to cope with anthropogenic stress. In October, there were no surviving shoots from *Ammophila* seeds in the first 50 m and no shoots from *Ammophila* rhizomes in the first 100 m. *Elytrigia* shoots were still present from both diaspores at these short distances from the path.

**Table 1:** Summary of the results of the smooth terms of the generalized additive model predicting the (interactive) effects of multiple variables on shoot counts per model for each monitoring moment. Significance codes: 0 = ***; 0.001 = **; 0.01 = *; 0.05 =. ; not significant = ns. The explained model deviance for May is 86.6%, for August 81.8%, and for October 73.4%

		Monitoring moment		
		May	August	October
Main variables	Path distance	ns	*	*
	Bed level change	***	**	*
	Sea distance	***	**	**
	Soil moisture	***	ns	ns
Interactions with path distance	Bed level change	ns	ns	ns
	Sea distance	**	ns	ns
	Soil moisture	*	ns	ns
Interactions path distance with treatments	*Ammophila arenaria* rhizomes	ns	.	ns
	*Ammophila arenaria* seeds	*	ns	ns
	*Elytrigia juncea* rhizomes	ns	ns	ns
	*Elytrigia juncea* seeds	ns	ns	ns
Interactions sea distance with treatments	*Ammophila arenaria* rhizomes	ns	ns	ns
	*Ammophila arenaria* seeds	**	ns	ns
	*Elytrigia juncea* rhizomes	ns	ns	ns
	*Elytrigia juncea* seeds	ns	ns	ns
Random factors	Study Area	**	*	.
	Block	***	***	***

### Interaction with environmental drivers

**Fig. 4:**
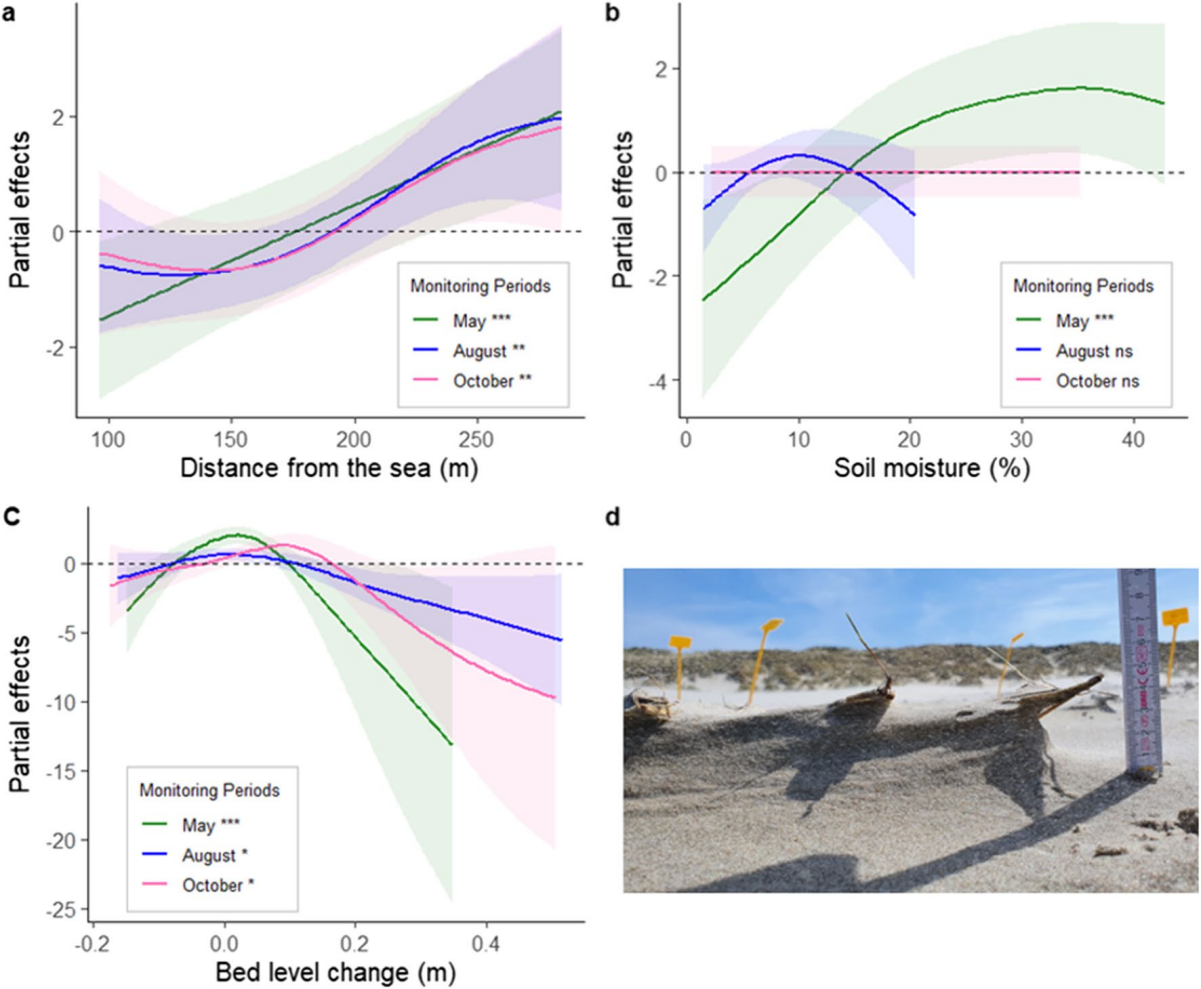
Modelled relationships of shoot number response by means of the partial effects of different smoothers relative to the mean (A) Sea distance (B) Soil moisture, and (C) Bed level change per monitoring moment with their confidence interval. In green: May, blue: August and pink: October. Above the horizontal dotted line at the 0 indicates a positive effect, below a negative effect of the variable. The shaded areas indicate the 95% confidence interval. (D) Rhizomes with approximately 6 cm of erosion

Environmental impacts on shoot numbers were additive to those of anthropogenic pressure for most moments: the only significant interactive effects with path distance were soil moisture and bed-level change in May (Table 1, Appendix Fig. 9). In general, bed level change ranged from − 0.17 to 0.52 m, soil moisture from 1.4 to 47.8% and sea distance from 96.4 to 284.0 m. Shoot numbers were affected by these environmental drivers irrespective of species and diaspore type (Fig. 4; Table 1, Appendix Figs. 6, 7 and 8). Bed level change and sea distance significantly affected shoot emergence and survival for all moments. On the other hand, the impact of soil moisture was only a significant contributor in May (Table 1). The smoother functions (Fig. 4) suggest non-linearity in shoot number responses to environmental drivers. Highest shoot numbers were associated with limited amount of bed-level change, further distances from the sea and high soil moisture. Responses across species and diaspore type were similar, with limited interactions. The only statistically significant interaction was between sea distance and *Ammophila* seeds in May, with more shoots emerging from seeds introduced further away from the sea (Table 1, Appendix Fig. 9).

## Discussion

In this study, we examined how anthropogenic pressure affects establishment of the dune-building grasses *Ammophila* and *Elytrigia*. We found that anthropogenic pressure, in the form of beach visitors constrains plant establishment irrespective of plant species or whether they establish from rhizomes or seeds. The effects can be regarded additive to those of environmental drivers, such as bed level change, soil moisture, and distance to the sea. The relative importance of these different drivers changes across the growing season (Figs. 3 and 4). While anthropogenic pressure affects establishment patterns starting during the tourism season, bed level change and sea distance play an important role during the entire growing season. Since dune-building plant establishment closely relates to the initial formation of coastal dunes, the above suggests that recreation adds to the spatial constrains of new dune development and thus co-determines the accommodation space.

### Plant success

In our field experiment only 5.6% of all introduced seeds and rhizomes showed shoot emergence from the soil. This is much lower than the shoot emergence reported for laboratory seed germination experiments, which range between 82 and 94% for *Ammophila* (Bonte et al. 2021; Huiskes 1979; Lim 2011) and 71–83% for *Elytrigia* (Del Vecchio et al. 2020). While establishment rates in the field may differ between plant populations and under different environmental conditions (Del Vecchio et al. 2022), the big difference between our field study and the lab studies is likely a result of the higher environmental stress in the field and the longer experimental period. Most lab germination studies optimise environmental conditions and typically last only a limited number of days focussing on seed germination only, limiting the time over which shoot mortality can occur. Seeds and rhizomes in our experiment were subjected to stressful field conditions for 9 months, including burial rates up to 52 cm. Given burial by sand strongly decreases shoot emergence from seeds and rhizomes (Lammers et al. 2024), our low values seem representative for field conditions.

We found small, but consistent differences in overall establishment success between dune building species, with *Elytrigia* (6.9%) being more successful than *Ammophila* (4.3%). These findings are in line with bare beach succession, as *Elytrigia* is observed earlier in the succession than *Ammophila* (Van Puijenbroek et al. 2017; van Puijenbroek, Limpens et al. 2017). Differences in plant responses to environmental drivers such as burial (Bonte et al. 2021; Harris and Davy 1986a; Lammers et al. 2024) and salinity (van Puijenbroek, Teichmann, et al. 2017) can also influence difference in success between the species. Although, the soil salinity levels during the plant growing period in our experiment did not exceed growth limiting levels (van Puijenbroek, Teichmann, et al. 2017).

The establishment from rhizomes (6.6%) was higher than from seeds (4.6%). A greater success of rhizome establishment over seeds has been documented (Harris and Davy 1986a; Van Der Putten 1990). Nevertheless, the interaction of species and diaspore showed that the success rates cannot only be attributed to species and diaspore specific responses. The treatments from high to low success were *Elytrigia* seeds, *Ammophila* rhizomes, *Elytrigia* rhizomes, and *Ammophila* seeds. For *Ammophila*, rhizomes do indeed perform better than seeds, which is in line with existing literature (Van Der Putten 1990). However, *Elytrigia* seeds outperform its rhizomes, similar to the findings by (Harris and Davy 1986a, b). The numbers in their study show seeds to have approximately 90% success of germinating while rhizomes have 70% shoot emergence. In addition, generally, larger seeds are more successful at germination and establishment than smaller ones (Moles and Westoby 2004). Based on their respective size and mass this might be an additional explanation of the higher success of *Elytrigia* seeds over those of *Ammophila*. Likewise, *Ammophila* rhizomes are larger, the difference in resource availability for the new plants might explain their higher overall emergence success rate than those from *Elytrigia*. Also, within species the allocation of energy can be observed in the relative size of the diaspores. Establishment success seems to be determined by the combination of both species and diaspore as well the effect of different drivers.

### Anthropogenic impact

Our study has shown decreased establishment of dune-building grasses near beach entrances during the period of highest recreation pressure for Dutch beaches (July-August). This effect was still significant in October and remained visible up to our last monitoring moment in January of the following year. While new shoots emerged from seeds and rhizomes continuously, the new recruitment was not enough to offset the negative impact of recreation. This illustrates the importance of including anthropogenic pressure as a driver of plant accommodation space, plant growth, and ultimately, dune development.

Juvenile plants are generally assumed to be more sensitive to disturbance than adult plants, as has been documented for both species in responses to burial (Bonte et al. 2021; Konlechner et al. 2013; Lim 2011; Nolet et al. 2018). Surprisingly, we find that the response to recreation pressure on new establishment of plants on beaches seem within the same order of magnitude as reported impacts on adult dune vegetation (Andersen 1995; Delgado-Fernandez et al. 2019; Farris et al. 2013; Poulson and Mcclung 1999; Šilc et al. 2017; Tzatzanis et al. 2003). However, these studies investigate the impact of recreation and trampling on pathways, and report localised negative impacts of trampling on or in the direct vicinity of these pathways. While at our study sites, visitors are not constrained to delineated pathways, but instead are able to move freely across the entire beach. The free movement likely reduces the localised impact, but rather enlarges the size of the affected area. This could explain why we find measurable negative effects on early plant establishment at a much further distance from the entrance (up to 100 m longshore) than expected from literature. This may also explain why a small fraction of our young plants still managed to persist under a much higher recreation pressure than the thresholds for survival previously reported for adult plants (Boorman and Fuller 1977; Burden and Randerson 1972; Hylgaard and Liddle 1981).

Although the statistical models have not shown a species or diaspore specific response to anthropogenic pressure, it can be noted that by October *Ammophila* had disappeared close to the main path (rhizomes absence < 100 m and seeds absence < 50 m distance from the most direct path from beach entrance to sea). In contrast, *Elytrigia* plants were still present close to the path. This trend suggests that *Ammophila* might be more sensitive to high anthropogenic pressure than *Elytrigia*. Indeed, other studies also report a relatively high sensitivity of adult *Ammophila* to trampling relative to other species (Boorman and Fuller 1977; Farris et al. 2013).

### Additive impact of environmental drivers

No significant interaction effects of anthropogenic pressure and environmental drivers were found for August and October, when anthropogenic pressure was a determining factor for plant establishment. This shows that anthropogenic impacts are additive to those of the environmental drivers and not caused by interactions between environmental drivers and anthropogenic pressure. In May, there was a significant interaction of anthropogenic pressure with soil moisture as well as with the proximity to the sea. This may hint at the relative importance of specific drivers at different moments across the growing season. However, for a conclusion about these interactions further research is required. Bed level change and distance to the sea were found to be important drivers for plant establishment success. Plant success was the highest under low levels of bed level change and seemed to favour burial over erosion. The maximum burial at which we found shoots from *Ammophila* seeds was 13 cm and from *Elytrigia* seeds 24 cm, which is higher than the 5–6 cm found in other studies for *Ammophila* (Bonte et al. 2021; Lim 2011; Maun 2009) and 17.8 cm for *Elytrigia* (Harris and Davy 1986a). However, in our study, sediment accretion and erosion were gradual and variable across the entire growing season, likely enabling the plants to adapt. Even though, the absence of major storm events between the set-up of our experiment and the first monitoring moment in May. The negative effect of proximity to the sea is likely related to the lower beach height near the sea and thus the increased exposure of inundation, salinity, and the exposure to wave action also during smaller high-water events (Nolet and Riksen 2019; van Puijenbroek, Limpenset al. 2017; van Puijenbroek, Teichmann et al. 2017). Given the rising sea level and increased likelihood of storm-related changes in bed-level (Oppenheimer et al. 2019), it is likely that the ability of upper beaches to support plant establishment will decrease in the future.

### Implications for dune-building and management

Taken together, our results suggest a trade-off between plant establishment and recreation at moderate recreation pressures. Given that coastal tourism is expected to increase with warmer temperatures (Coombes and Jones 2010), this suggests that recreation may reduce the beach area suitable for establishment of dune building grasses and associated future embryo dune establishment. Insufficient accommodation space for embryo dune development does not only impact biodiversity on beaches but may also leave the base of the foredunes (the dune toe) more vulnerable to storm erosion. For this reason, facilitating both recreation and dune development at any location will require a delicate balance. Our findings suggest the impact of anthropogenic pressure is associated with the dominant route people follow from beach entrance towards the sea, which in our case seemed to follow a funnel shape, with the funnel widening nearing the sea. Thus, measures aimed at directing visitor flows over the upper beach may limit the effects of trampling. A potential option may be to direct visitors with semi-constructed pathways rather than letting them roam freely across the upper beach. Directing people can be done in several ways, in Italy it has been shown that the introduction of boardwalks to direct visitors in the larger dune area has reduced trampling and resulted in increased species richness and vegetation cover (Prisco et al. 2021). Fencing (either with wire or rushes) is a much-seen option in the Netherlands at the foredune to create pathways to guide visitors. It might be possible to extend these measures further onto beaches to help localising anthropogenic pressures, thus supporting embryonic dune development and associated biodiversity while still accommodating recreation.

## Conclusion

In this study we showed that anthropogenic pressure has a negative impact on the establishment of dune-building grasses on the upper beach. This is the case for plant establishment from seeds and rhizomes of both *Ammophila* and *Elytrigia*. Both environmental factors and anthropogenic pressure are drivers of the accommodation space for dune vegetation establishment, with the relative importance of these drivers differing with the season. Together, this suggests a trade-off between the ecosystem services of recreation, and those of biodiversity and new dune development. This trade-off will require careful navigation when managing and designing multifunctional beaches.

## Figures and Tables

**Fig. 5:**
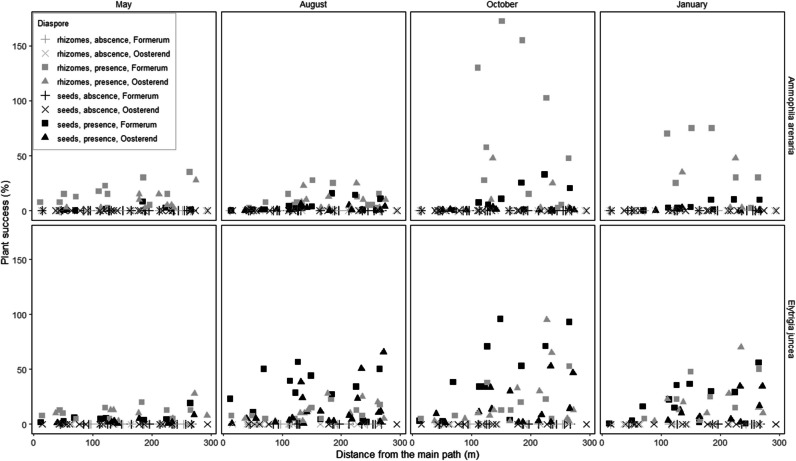
Relative plant success per plot (relative to the number of introduced diaspores) vs path distance per monitoring period per study area for all treatments (diaspores x species), absence and presence of plants per plots are indicated for visual clarity in the lower success range

**Fig. 6:**
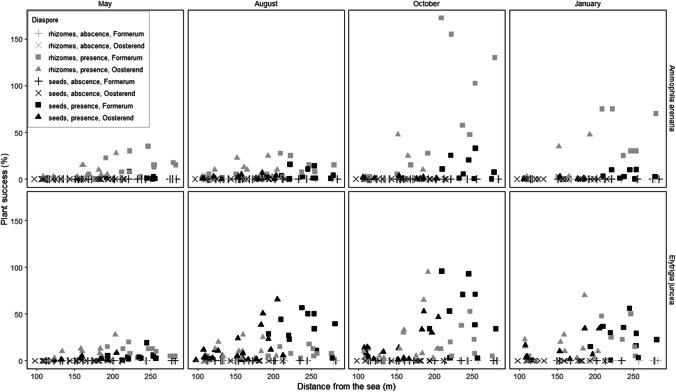
Relative plant success per plot (relative to the number of introduced diaspores) vs sea distance per monitoring period per study area for all treatments (diaspores x species), absence and presence of plants per plots are indicated for visual clarity in the lower success range

**Fig. 7:**
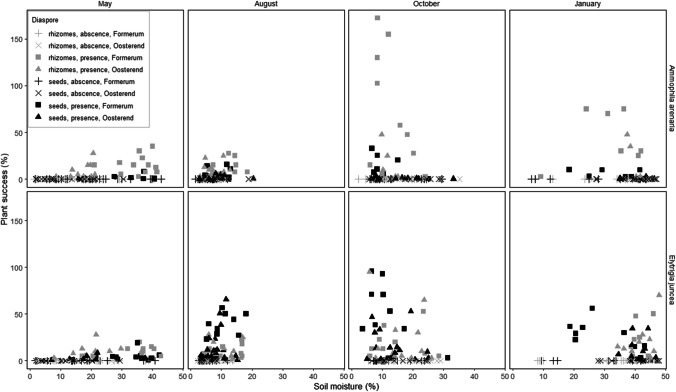
Relative plant success per plot (relative to the number of introduced diaspores) vs soil moisture per monitoring period per study area for all treatments (diaspores x species), absence and presence of plants per plots are indicated for visual clarity in the lower success range

**Fig. 8:**
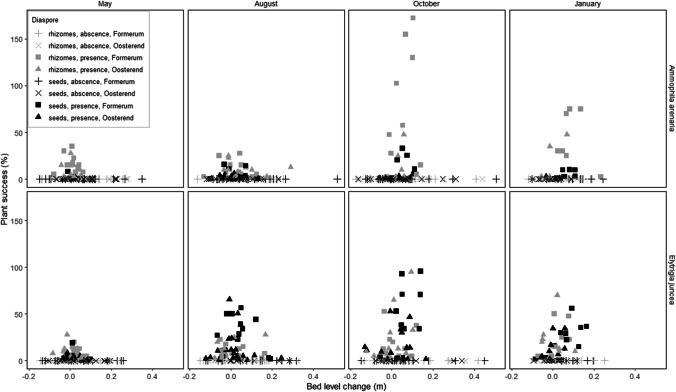
Relative plant success per plot (relative to the number of introduced diaspores) vs bed level change per monitoring period per study area for all treatments (diaspores x species), absence and presence of plants per plots are indicated for visual clarity in the lower success range

**Fig. 9:**
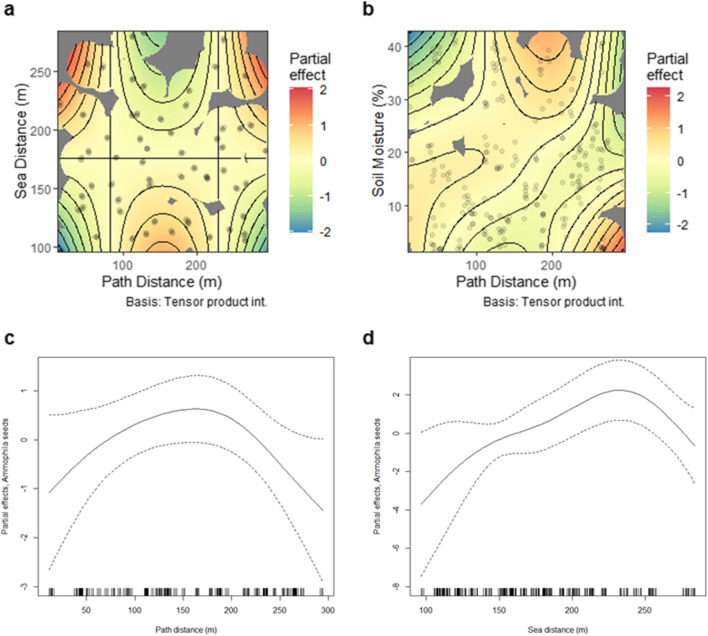
Significant interaction with path distance in May. A) Interaction with soil moisture and B) interaction with sea distance. The colour indicate the partial effects, above 0 this is positive effect on shoot numbers and below 0 a negative effect. C) Interaction with *Ammophila* seeds and D) interaction of sea distance with *Ammophila* seeds. Above the horizontal dotted line at the 0 indicates a positive effect, below a negative effect of the variable.

## Appendix

Equation (2)

2 $$Model\;=\;gam\left(Plant\;Number^\sim\;Treatment\;+\;s\left(BedLevelChange\right)\;+\;s\left(Moisture\right)\;+\;s\left(PathDis\tan ce\right)\;+\;s\left(PathDis\tan ce,\;by\;=Treatment\right)\;+\;s\left(SeaDis\tan ce\right)\;+\;s\left(SeaDis\tan ce,\;by\;=Treatment\right)\;+\;ti\left(PathDis\tan ce,\;SeaDis\tan ce\right)\;+\;ti\left(PathDis\tan ce,\;BedLevelChange\right)\;+\;ti\left(PathDis\tan ce,\;Moisture\right)\;+\;s\left(StudyArea,\;bs\;=\;'re'\right)\;+\;s\left(Block,\;bs\;=\;'re'\right),\;data\;=\;Terschelling\lbrack Terschelling\$MonitoringPeriod\;==\;1,\rbrack,\;method\;=\;'REML',\;select\;=\;TRUE,\;family\;=\;nb\;()\;\right)$$

Tables 2, 3, 4, 5, 6, 7 and Figs. 5, 6, 7, 8, 9

**Table 2:** Mean relative plant success percentage with standard error per treatment for each monitoring period, significances are tested on the overall success with a Kruskal-Wallis, chi-squared = 25.302, df = 3, p-value = 1.335e-05, and Wilcoxon rank sum test

	May	August	October	January	Overall
*Ammophila arenaria* rhizomes	4.5 ±0.14	4.0 ±0.12	13.4 ±0.61	8.6 ±0.44	7.3^b^ ±0.10
*Ammophila arenaria* seeds	0.2 ±0.02	1.3 ±0.05	1.9 ±0.10	0.9 ±0.05	1.1^a^ ±0.02
*Elytrigia juncea* rhizomes	3.3 ±0.10	4.0 ±0.12	7.6 ±0.33	7.9 ±0.05	5.6^b,c^ ±0.05
*Elytrigia juncea* seeds	1.2 ±0.05	11.0 ±0.29	12.4 ±0.40	8.0 ±0.30	8.2^c^ ±0.07

**Table 3:** Pairwise comparison using Wilcoxon rank sum test of visitor counts per distance group for Formerum. Kruskal-Wallis chi-squared = 45.585, df = 2, p-value = 1.263e-10

Path distance	0-100	100-200
100-200	2.6e-07	-
200-300	2.0e-08	0.0035

**Table 4:** Pairwise comparison using Wilcoxon rank sum test of visitor counts per distance group for Oosterend. Kruskal-Wallis chi-squared = 42.141, df = 2, p-value = 7.065e-10

Path distance	0-100	100-200
100-200	7.4e-05	-
200-300	8.2e-08	1.6e-05

**Table 5:** Model summary for plant number during the May monitoring moment

Component	Term	Estimate	Std Error	z-value	p-value	
A. parametric coefficients	(Intercept)	-1.98	1.01	-1.97	0.05	*
	TreatmentAa se	-1.77	0.65	-2.74	0.01	**
	TreatmentEj rhi	-0.36	0.29	-1.22	0.22	
	TreatmentEj se	0.11	0.32	0.36	0.72	
Component	Term	edf	Ref.df	Chi-sq	p-value	
B. smooth terms	s(SedimentChange)	3.41	9.00	35.25	0.00	***
	s(Moisture)	2.64	9.00	42.75	0.00	***
	s(PathDistance)	0.00	9.00	0.00	0.84	
	s(PathDistance):TreatmentAa rhi	0.00	9.00	0.00	0.68	
	s(PathDistance):TreatmentAa se	1.86	9.00	5.42	0.04	*
	s(PathDistance):TreatmentEj rhi	0.00	9.00	0.00	0.64	
	s(PathDistance):TreatmentEj se	0.00	9.00	0.00	0.91	
	s(SeaDistance)	0.94	9.00	38.75	0.00	***
	s(SeaDistance):TreatmentAa rhi	0.00	9.00	0.00	0.89	
	s(SeaDistance):TreatmentAa se	2.77	9.00	12.79	0.00	**
	s(SeaDistance):TreatmentEj rhi	0.00	9.00	0.00	0.55	
	s(SeaDistance):TreatmentEj se	0.70	9.00	2.22	0.08	.
	ti(PathDistance,SeaDistance)	1.52	16.00	13.19	0.00	**
	ti(PathDistance,SedimentChange)	0.87	16.00	2.39	0.12	
	ti(PathDistance,Moisture)	3.90	16.00	18.74	0.02	*
	s(StudyArea)	0.91	1.00	16.88	0.00	**
	s(BlockSA)	12.95	59.00	23.39	0.00	***

Signif. codes: 0 <= '***' < 0.001 < '**' < 0.01 < '*' < 0.05

Adjusted R-squared: 0.587, Deviance explained 0.864

-REML : 263.952, Scale est: 1.000, N: 240

**Table 6:** Model summary for plant number during the August monitoring moment

Component	Term	Estimate	Std Error	z-value	p-value	
A. parametric coefficients	(Intercept)	-1.11	0.71	-1.55	0.12	
	TreatmentAa se	1.47	0.31	4.67	0.00	***
	TreatmentEj rhi	0.11	0.34	0.32	0.75	
	TreatmentEj se	2.57	0.30	8.49	0.00	***
Component	Term	edf	Ref.df	Chi-sq	p-value	
B. smooth terms	s(SedimentChange)	2.69	9.00	173.39	0.00	**
	s(Moisture)	1.82	9.00	20.43	0.21	
	s(PathDistance)	1.47	9.00	91.08	0.02	*
	s(PathDistance):TreatmentAa rhi	1.39	9.00	3.77	0.07	.
	s(PathDistance):TreatmentAa se	0.00	9.00	0.00	0.74	
	s(PathDistance):TreatmentEj rhi	0.00	9.00	0.00	0.56	
	s(PathDistance):TreatmentEj se	0.00	9.00	0.00	0.71	
	s(SeaDistance)	1.83	9.00	330.82	0.00	**
	s(SeaDistance):TreatmentAa rhi	0.00	9.00	0.00	0.54	
	s(SeaDistance):TreatmentAa se	0.64	9.00	0.95	0.21	
	s(SeaDistance):TreatmentEj rhi	0.00	9.00	0.00	0.46	
	s(SeaDistance):TreatmentEj se	0.00	9.00	0.00	0.60	
	ti(PathDistance,SeaDistance)	0.00	16.00	0.00	0.64	
	ti(PathDistance,SedimentChange)	0.00	16.00	0.00	0.57	
	ti(PathDistance,Moisture)	0.00	16.00	0.00	0.71	
	s(StudyArea)	0.80	1.00	70.07	0.05	*
	s(BlockSA)	41.42	59.00	153.80	0.00	***

Signif. codes: 0 <= '***' < 0.001 < '**' < 0.01 < '*' < 0.05

Adjusted R-squared: 0.571, Deviance explained 0.818

-REML : 505.660, Scale est: 1.000, N: 240

**Table 7:** Model summary for plant number during the October monitoring moment

Component	Term	Estimate	Std Error	z-value	p-value	
A. parametric coefficients	(Intercept)	-1.26	0.86	-1.46	0.14	
	TreatmentAa se	1.13	0.49	2.33	0.02	*
	TreatmentEj rhi	0.57	0.50	1.14	0.26	
	TreatmentEj se	2.25	0.47	4.74	0.00	***
Component	Term	edf	Ref.df	Chi-sq	p-value	
B. smooth terms	s(SedimentChange)	3.55	9.00	54.05	0.04	*
	s(Moisture)	0.00	9.00	0.00	0.44	
	s(PathDistance)	1.47	9.00	38.04	0.03	*
	s(PathDistance):TreatmentAa rhi	1.64	9.00	2.90	0.17	
	s(PathDistance):TreatmentAa se	0.00	9.00	0.00	0.80	
	s(PathDistance):TreatmentEj rhi	0.00	9.00	0.00	0.56	
	s(PathDistance):TreatmentEj se	0.00	9.00	0.00	0.99	
	s(SeaDistance)	1.69	9.00	198.00	0.01	**
	s(SeaDistance):TreatmentAa rhi	0.00	9.00	0.00	0.80	
	s(SeaDistance):TreatmentAa se	0.00	9.00	0.00	0.44	
	s(SeaDistance):TreatmentEj rhi	0.00	9.00	0.00	0.73	
	s(SeaDistance):TreatmentEj se	0.00	9.00	0.00	0.71	
	ti(PathDistance,SeaDistance)	0.00	16.00	0.00	0.97	
	ti(PathDistance,SedimentChange)	0.00	16.00	0.00	0.72	
	ti(PathDistance,Moisture)	0.00	16.00	0.00	0.98	
	s(StudyArea)	0.78	1.00	27.08	0.06	.
	s(BlockSA)	36.78	59.00	111.07	0.00	***

Signif. codes: 0 <= '***' < 0.001 < '**' < 0.01 < '*' < 0.05

Adjusted R-squared: 0.436, Deviance explained 0.728

-REML : 483.042, Scale est: 1.000, N: 240
